# Molecular profiles and immunomodulatory activities of glioblastoma-derived exosomes

**DOI:** 10.1093/noajnl/vdaa056

**Published:** 2020-05-06

**Authors:** Juliana Hofstatter Azambuja, Nils Ludwig, Saigopalakrishna Yerneni, Aparna Rao, Elizandra Braganhol, Theresa L Whiteside

**Affiliations:** 1 Postgraduate Program in Biosciences, Federal University of Health Sciences of Porto Alegre (UFCSPA), Porto Alegre, Brazil; 2 Department of Pathology, University of Pittsburgh School of Medicine, Pittsburgh, Pennsylvania, USA; 3 UPMC Hillman Cancer Center, Pittsburgh, Pennsylvania, USA; 4 Department of Biomedical Engineering, Carnegie Mellon University, Pittsburgh, Pennsylvania, USA; 5 Department of Neurological Surgery, University of Pittsburgh School of Medicine, Pittsburgh, Pennsylvania, USA; 6 Departments of Immunology and Otolaryngology, University of Pittsburgh School of Medicine, Pittsburgh, Pennsylvania, USA

**Keywords:** exosomes, glioblastoma, immune system, macrophages, tumor microenvironment

## Abstract

**Background:**

Glioblastoma is one of the most immunosuppressive human tumors. Emerging data suggest that glioblastoma-derived exosomes (GBex) reprogram the tumor microenvironment into a tumor-promoting milieu by mechanisms that not yet understood.

**Methods:**

Exosomes were isolated from supernatants of glioblastoma cell lines by size exclusion chromatography. The GBex endosomal origin, size, protein cargos, and ex vivo effects on immune cell functions were determined. GBex were injected intravenously into mice to evaluate their ability to in vivo modulate normal immune cell subsets.

**Results:**

GBex carried immunosuppressive proteins, including FasL, TRAIL, CTLA-4, CD39, and CD73, but contained few immunostimulatory proteins. GBex co-incubated with primary human immune cells induced simultaneous activation of multiple molecular pathways. In CD8^+^ T cells, GBex suppressed TNF-α and INF-γ release and mediated apoptosis. GBex suppressed natural killer (NK) and CD4^+^ T-cell activation. GBex activated the NF-κB pathway in macrophages and promoted their differentiation into M2 cells. Inhibition of the NF-κB pathway in macrophages reversed the GBex-mediated effects. GBex-driven reprogramming of macrophages involved the release of soluble factors that promoted tumor proliferation in vitro. In mice injected with GBex, the frequency of splenic CD8^+^ T cells, NK cells, and M1-like macrophages was reduced, while that of naïve and M2-like macrophages increased (*P* < .05).

**Conclusions:**

GBex reprogrammed functions of all types of immune cells in vitro and altered their frequency in vivo. By creating and sustaining a highly immunosuppressive environment, GBex play a key role in promoting tumor progression.

**Key Points**

- GBex are enriched in inhibitory proteins and suppress the functions of all types of immune cells.
- GBex reprogram immune cells in vitro and in vivo, creating systemic immunosuppression.
- Immune cells reprogrammed by GBex promote glioblastoma progression.

**Importance of the Study**

This study examines the molecular underpinnings of the immunosuppressive activity of GBex. It shows that GBex were enriched in inhibitory proteins which dramatically suppressed immune cell activities in vitro. GBex carried few immunostimulatory proteins. GBex delivered multiple inhibitory signals to all types of immune cells, *simultaneously* activating several different molecular pathways. Macrophages, which avidly internalized GBex, were highly susceptible in vitro and in vivo to GBex-mediated reprogramming. FasL-induced activation of the NF-κB pathway in macrophages was instrumental for M1 polarization to M2, and blocking of NF-κB signaling reversed the M2 phenotype to M1. GBex also promoted glioblastoma progression in vitro. Injected into normal mice, GBex decreased the frequency of CD8^+^ T cells and M1 macrophages, increasing that of M2 cells in the spleen. Protecting immune cells, especially CD8^+^ T cells and M1 macrophages, from GBex-mediated effects emerges as a potential therapeutic target for future immunotherapy of glioblastoma.

Every year, 100 000 people are diagnosed with gliomas worldwide.^1^ The World Health Organization (WHO) classifies gliomas into the pathologic grades I–IV, and the majority of patients develop the most malignant grade IV disease, called glioblastoma (GB).^2^ The standard therapy includes surgery, followed by chemo- and radiotherapy.^3^ However, treatment of brain tumors remains a challenge, largely due to their biological characteristics, including high proliferation rate, aggressive tissue infiltration, development of chemoresistance, increased angiogenesis, as well as the structural complexity of the brain, the presence of blood–brain barrier (BBB), and profound changes that take place in the tumor microenvironment (TME).^4^ GB remains essentially incurable, with overall survival ranging from 12 to 14 months.^5^ Fewer than 5% of patients survive longer than 5 years.^6^ Clearly, novel approaches to GB therapy are an urgent, and so far, unmet need.

The TME of GB includes non-cancerous cells present inside the tumor mass, including astrocytes, neurons, cancer stem cells, fibroblasts, immune cells, microglia/macrophages, and endothelial cells.^7^ In malignant gliomas, the mixture of microglia/macrophages can comprise up to one-third of the tumor mass.^8^ The dilemma of whether this subset of cells represents “friends or foes” has not been completely resolved, but a growing body of evidence emphasizes their propensity for phenotypical transformation into tumor-associated macrophages (TAMs). TAMs act directly to promote GB growth and to create an immunosuppressive TME.^9,10^ Additionally, M2-polarized TAMs mediate immunosuppression by altering functions or survival of other immunocytes.^11,12^ For example, M2-like macrophages express Fas ligand (FasL), and induce apoptosis of activated T cells^13^ and promote Treg formation.^14^

The GB-derived extracellular vesicles (EVs) might represent one of the mechanisms contributing to immunosuppression in the TME and making it more permissive for cancer progression.^15^ Among EVs, exosomes represent small (30–150 nm) vesicles that originate from the endocytic compartment of parental cells and mimic the content and functions of parental cells. Exosomes are enclosed in a lipid/protein bilayer membrane surrounding an aqueous core of numerous soluble proteins and nucleic acids.^16^

Glioblastoma-derived exosomes (GBex) obtained from patients’ plasma were previously reported to induce suppression of T-cell proliferation and cytokine production.^17^ However, other data in the literature suggest that patient-derived GBex promote expression of immunosuppressive phenotypes in monocytes but do not alter functions of cytotoxic T cells, implying that GBex preferentially target myeloid cells.^18,19^ GBex were also reported to enhance differentiation of myeloid-derived suppressor cells (MDSCs) and to upregulate TGF-β and IL-10 expression in MDSCs.^20–23^ In this report, we use GBex produced by GB cell lines to examine GBex interactions with primary human immune cells in vitro and in vivo. Our data show that GBex mediate extensive molecular reprogramming of all types of immune cells that leads to their functional paralysis and promotes tumor progression.

## Materials and Methods

### Cell Lines

U87MG, SBN19, and U251 human GB cell lines were obtained from ATCC and were grown in Dulbecco’s modified Eagle’s medium (Gibco Fisher Scientific) containing 10% (v/v) exosome-depleted and heat-inactivated fetal bovine serum (FBS) (Gibco Fisher Scientific). The CD8^+^ Jurkat cell line was obtained from Dr H. Rabinowich (Department of Pathology, University of Pittsburgh, PA)^24^ and grown in RPMI containing 10% (v/v) exosome-depleted and heat-inactivated FBS. Cells were kept at 37°C in a humidified atmosphere of 5% CO_2_ in the air. For exosome isolation, 4 × 10^6^ cells were cultured in 150 cm^2^ culture flasks containing 25 mL of culture medium. After 72 h, supernatants were collected and used for exosome isolation.

### Peripheral Blood Mononuclear Cells

Blood samples were obtained from healthy donors and peripheral blood mononuclear cells (PBMCs) were isolated by Ficoll Paque Plus (GE Healthcare Bioscience) by gradient centrifugation as previously described.^25^ All subjects donating blood specimens signed an informed consent approved by the Institutional Review Board of the University of Pittsburgh (IRB #960279 and IRB #0506140). PBMCs were used for the isolation of CD4^+^ T, CD8^+^ T, and NK cells by negative selection using AutoMACS (Miltenyi) with the cell isolation kits (REF 130-096-533, 130-096-495, and 130-092-657, respectively) as previously described.^25^ Monocytes were separated by adherence to plastic. Isolated cells were cultured in RPMI containing 10% (v/v) exosome-depleted and heat-inactivated FBS.

### Exosome Isolation by Mini-SEC

Exosomes were isolated by size exclusion chromatography (SEC) as previously described.^26^ Exosomes were collected in fraction #4 (1 mL). For some experiments, #4 mini-SEC fractions were concentrated to 1 µg/µL using 100 000 MWCO Vivaspin 500 Centrifugal Concentrators (Sartorius Corp, #7321011) at 5000 × *g* for 5–10 min. Protein contents were measured using the BCA protein assay kit (Thermo Scientific, REF 23225). Transmission electron microscopy (TEM), tunable resistive pulse sensing measurements, and western blots were used to determine morphology, size, particle concentration, and protein cargo of exosomes as previously described by us.^27^

### RAW-Blue Assay

RAW-Blue cells (a murine RAW 264.7 macrophage reporter cell line) were purchased from InvivoGen. This reporter cell line stably expresses a secreted embryonic alkaline phosphatase gene inducible by NF-κB activation that can be detected. The assay was performed according to the manufacturer’s instructions. Briefly, 2 × 10^4^ RAW-blue cells were incubated with 20 µg of GBex alone or in combination with (1) NF-κB pathway inhibitor (IKK16, 200 nmol, 2539, Tocris), (2) anti-TGF-β Ab (IDII, 100 nM, obtained from Dr Andrew Hinck, University of Pittsburgh), (3) COX inhibitor (Celecoxib, 1 µM, PZ0008-, Sigma), (4) anti-IL-2R Ab (10 µg, MAB224, Millipore), (5) CD73 inhibitor (Adenosine 5′-(α,β-methylene)diphosphate, 1 µM, M3763, Sigma), (6) phosphodiesterase inhibitor (Rolipram, 1 µM, R6520, Sigma), (7) anti-IL-6 Ab (10 µg, mabg-hil6-3, Invivogen), (8) anti-FAS Ab (10 µg, 05-338, Millipore), and (9) anti-TNF-α Ab (10 µg, htnfa-mab1, Invivogen) for 24 h under standard conditions. After incubation, 20 µL of media was collected and incubated with 200 µL QUANTI-blue reagent (Invivogen) and optical density was measured at 655 nm using TECAN spectrophotometer (TECAN).

### Exosome Immunoregulatory Activities Measured In Vitro

#### Uptake of labeled GBex by T-cell subsets or macrophages.

—U251 GBex were labeled with the SYTO RNASelect Green Fluorescent cell dye according to the manufacturer’s instructions (S32703, Thermo Fisher). Labeled exosomes (10 μg protein) were co-incubated with 2 × 10^5^ primary CD8^+^ T cells, CD4^+^ T cells, or macrophages for 0.25, 0.5, 1, 6, 24, 48, and 72 h at 37°C. Cells were washed 3× with PBS and immediately analyzed in an Accuri flow cytometer (BD Biosciences).

#### Annexin V-based apoptosis of CD8+ primary T cells or Jurkat cells.

—CD8^+^ primary activated T cells or Jurkat cells were pre-plated (10^5^ cells/well in a 48-well plate) in RPMI for 6 h. Next, freshly prepared GBex (1, 2.5, 5, 10, and 20 μg) were added to the wells and co-incubated for 24 h. Cultures without exosomes but with the same volume of PBS served as controls. Apoptosis of CD8^+^ T cells was measured using FITC Annexin V Apoptosis Detection Kit (556547, BD Biosciences) according to the manufacturer’s instructions using an Accuri flow cytometer (BD Biosciences).

#### MTS assay with Jurkat cells.

—Jurkat cells were seeded and plated as described above. The MTS cell viability assay was performed according to the manufacturer’s instructions (ab197010, Abcam). Cell viability was calculated using Prism 7.0 software (Prism GraphPad Software) according to the following formula: cell viability rate (%) = (OD490 of treated cells/OD490 of control) × 100% and cells incubated with PBS served as controls. For the Ab blocking experiments, Jurkat cells were incubated with anti-FAS Ab (10 µg, 05-338 lot#2896737 clone ZB4, Millipore) and an appropriate isotype control for 1 h before the addition of exosomes.

#### Flow cytometry with CD8+ Jurkat cells.

—Jurkat cells were plated as described above. After 24 h of incubation with various concentrations of GBex (1–20 µg), immunomodulatory molecules expression in lymphocytes was evaluated by flow cytometry. Cells were washed with PBS and fixed with eBioscience IC Fixation Buffer (00-8222-49) for 20 min at room temperature (RT). Cells were then permeabilized with eBioscience Permeabilization Buffer (00-8333-56) and incubated with specific antibodies (Supplementary Table 1) for 30 min in a blocking solution at RT in the dark with a minimum of 2 washes with PBS after antibody incubation. Cell fluorescence was measured with an Accuri flow cytometer (BD Biosciences).

#### CD69 downregulation in CD4+ T cells.

—Human primary CD4^+^ T cells were activated using anti-CD2/anti-CD3/anti-CD28 beads (130-091-441, Miltenyi) at the 1:2 beads to cell ratio in the presence of IL-2 (150 U/mL, Peprotech) overnight. GBex (50 μg) were co-cultured with activated CD4^+^ T cells (1 × 10^5^ cells in 200 µL in a 48-well plate) in 10% RPMI for 48 h. Changes in CD69 expression levels on T cells were measured by flow cytometry. Controls included isotype Abs, resting/nonactivated T cells, and activated T cells alone.

#### NKG2D expression in NK cells.

—Human NK cells were isolated and pre-plated (10^5^cells/well in a 48-well plate) in RPMI. The cells were activated with IL-2 (100 U/mL, Peprotech) overnight prior to incubation with exosomes. Afterward, NK cells were co-incubated with exosomes (25 μg protein/2 × 10^6^ cells/1 mL) for 48 h, stained and analyzed for NKG2D expression using flow cytometry.

### Macrophage Characterization

Macrophages were generated from primary human monocytes as previously described.^28^ After differentiation, control macrophages (Mϕ Naive) were maintained in RPMI. GBex (25μg protein/2 × 10^6^ cells/1 mL) were co-cultured with macrophages for 72 h. Macrophages were phenotyped by flow cytometry. In experiments with the NF-κB pathway inhibitor, macrophages were exposed to IKK16 (200 nmol, 2539, Tocris) for 30 min prior to the addition of GBex.

To study macrophages polarization, changes in expression of M1/M2 markers on macrophages were measured by flow cytometry after surface or intracellular staining with specific antibodies (Supplementary Table 1). Cell fluorescence was measured with an Accuri flow cytometer (BD Biosciences).

### Phosphokinase Array

The relative levels of phosphorylated kinases in macrophages and CD8^+^ T cells co-incubated with GBex were measured using a Phosphokinase Array Kit (R&D Systems Inc.). Aliquots of proteins (300 μg) isolated from lysed cells were added to the array, and the results were analyzed using the ImageJ software (http://rsbweb.nih.gov/ij/).

### Indirect Effects of GBex on Macrophages

To prepare macrophage conditioned medium (CM), cells were plated and cultured with GBex as described above. After 72 h the medium was collected, centrifuged (1000 × *g* for 10 min), and stored at −80°C until use. U251 cells were seeded in wells of 96-well plates and incubated with 100 µL fresh in a medium for 24 h. Aliquots (100 µL) of CM were added to all wells and after 48 h the cell viability was evaluated with MTS assay. Cells cultured in fresh medium or in CM from naïve macrophages were used as controls.

### Immunoregulatory Activities of GBex In Vivo

This study was carried out in strict accordance with the recommendations in the Guide for the Care and Use of Laboratory Animals of the NIH. The protocol (18042580) was approved by the Institutional Animal Care and Use Committee of the University of Pittsburgh (Animal Welfare Assurance Number: D16-00118). Normal male C57BL/6 mice (25–30 g, 6 weeks old) were purchased from The Jackson Laboratory. Animals were randomly divided into 2 groups as follows (n = 7): (1) PBS-treated (Control) and (2) GBex-treated. GBex were administered intravenously, every 3 days at doses of 1 mg/kg for 16 days. The control group received the equivalent volumes of PBS. At the end of the protocol, mice were euthanized, and blood and spleen collected (see details in Supplementary Figure 6).

Spleens were placed in RPMI and single-cell suspensions were prepared by mechanically pressing cells through a 70 μm cell-strainer. PBMCs were isolated from blood. Cells were stained with the labeled antibodies (Supplementary Table 1) for 60 min at RT in the dark. The data were acquired on LSR Fortessa Flow cytometer (BD Biosciences) and analyzed using the Flow Jo software.

### Statistical Analysis

Statistics were performed using GraphPad Prism 7 software; data were expressed as mean ± SD and subjected to one-way analysis of variance followed by Tukey–Kramer post hoc test for multiple comparisons or Student’s *t*-test. Differences were considered significant at *P < .05.

## Results

### Exosome Isolation and Characterization

**Figure 1.**
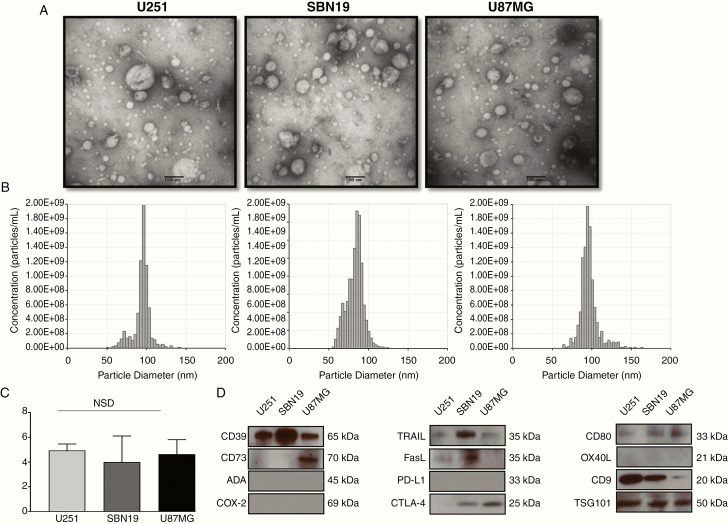
Characteristics and the immunomodulatory profile of GBex. GB cells were plated and expanded for 72 h. Cell supernatants were harvested, pre-cleared, concentrated to 1 mL, and used for exosome isolation by mini-SEC. (A) Representative transmission electron microscopy images of GBex produced by the 3 GB cell lines (U87MG, SBN19, and U251). (B) qNano analyses of the isolated GBex. (C) Total protein levels isolated from supernatants of GB cells. The data are mean values ± SD from 3 experiments. Data were analyzed by ANOVA followed by Tukey post hoc. (D) Western blot profiles of GBex isolated from the supernatants of the GB cell lines. Each lane was loaded with 10 μg GBex protein. Note the presence of exosome markers (CD9 and TGS101) and the immunoinhibitory proteins (CD39, CD73, FasL, CTL-4, TRAIL).

Exosomes were isolated from supernatants of GB cell lines by mini-SEC as described by us^27^ and vesicles in fraction #4 were harvested. TEM showed that vesicles isolated from supernatants of 3 different GB cell lines were similar in size, ranging from 30 to 150 nm in diameter, and vesicular morphology (Figure 1A and B). This places them in the category of small EVs. The presence of TSG101 in the vesicle cargo confirmed their origin from the endocytic compartment of parental cells, suggesting they are exosomes (Figure 1C). The isolated exosomes contained from 2 to 6 μg protein/10^6^ GB cells. There were no significant differences in the levels of total exosome protein recovered from the 3 GB cell lines (U87MG—4.3 μg/10^6^ cells; U251—3.99 μg/10^6^ cells, and SBN19—4.63 μg/10^6^ cells).

Exosomes produced by the GB cell lines carried CD39, CD73, FasL, CTLA-4, and TRAIL, proteins that are known to mediate immunosuppression. Exosomes were negative for adenosine deaminase, PDL-1, COX-2, and OX40L and weakly positive for CD80. This initial profiling suggested that immunosuppressive rather than immunostimulatory proteins were the main cargo components of GBex (Figure 1C).

**Figure 2.**
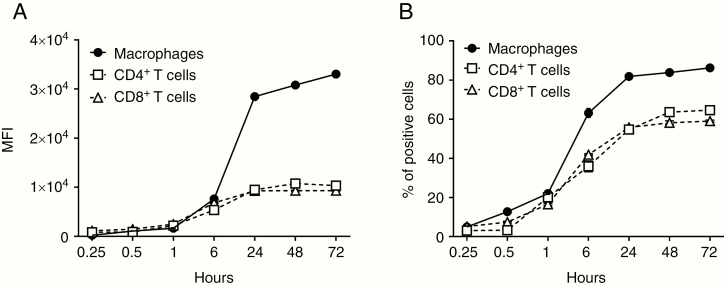
Uptake of labeled GBex by cells. Human macrophages, CD4^+^ T and CD8^+^ T cells (1 × 10^5^ cells in wells of a 48-well plate) were co-incubated with GBex (10 µg) isolated from supernatants of U251 GB cell line and labeled with SYTO RNASelect Green Fluorescent cell stain dye as described in Methods. Flow cytometry was performed at the indicated time points, and the graphs show the mean fluorescence intensity (MFI) in (A) and % of positive cells in (B).

GBex were labeled with SYTO RNASelect Green Fluorescent cell stain dye and co-incubated with human primary activated CD4^+^ T, CD8^+^ T cells, or macrophages. These cells internalized labeled GBex, although macrophages did so rapidly and more efficiently than T cells. At 24–72 h of incubation, macrophages internalized 3.5-fold more exosomes than did T cells. After 72 h, 86% of macrophages were positive for exosomes versus only 64% of CD4^+^ T cells and 59% of CD8^+^ T cells (Figure 2A and B).

### GBex Modulate Functions of T Cells and NK Cells

Human primary CD4^+^ T cells or NK cells were co-incubated with GBex (25 µg/2 × 10^6^ cells/mL) for 24 h. As shown in Supplementary Figure 1A, GBex suppressed CD69 expression levels and CD4^+^ T-cell activation. GBex co-incubated with primary human-activated NK cells suppressed NKG2D expression levels and thus NK activation (Supplementary Figure 1B). However, GBex had no effect on Treg expansion (data not shown).

**Figure 3.**
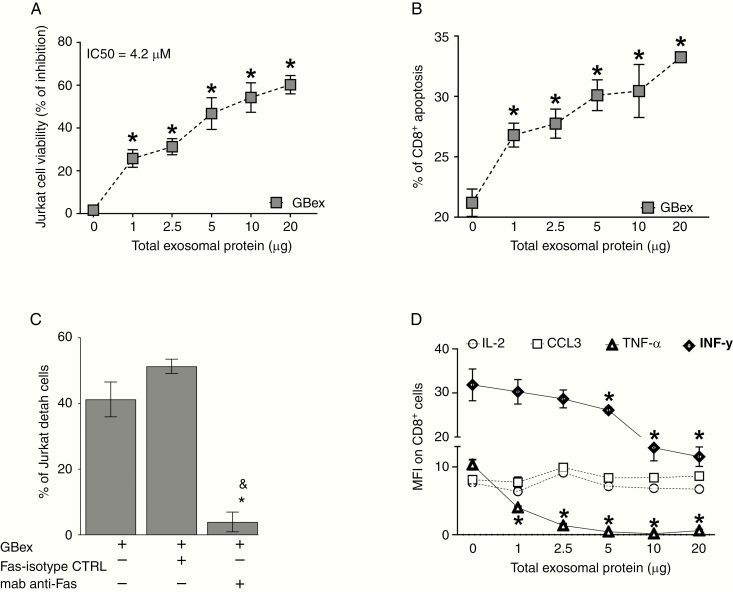
GBex induced apoptosis of primary CD8+ T cells and reduced cytokine expression. (A) Cell viability: CD8^+^ Jurkat cells were co-incubated for 24 h with increasing protein levels of GBex isolated from supernatants of the U251 GB cell line. (B) Annexin V-stained primary human-activated CD8^+^ T cells incubated with GBex or PBS as a control for 24 h. (C) Cell viability of CD8^+^ Jurkat cells preincubated with anti-FAS Mab and after 1 h with 4.2 µg of GBex protein isolated from supernatants of the U251 GB cell line. *Significantly different from control cells and ^&^different from FAS-isotype control (CTRL). (D) Expression levels of IL-2, CCL3, TNF-α, and INF-γ measured by flow cytometry in CD8^+^ Jurkat cells co-incubated with GBex. Data were analyzed by ANOVA followed by post hoc comparisons (Tukey test). *Significantly different from control cells at *P* < .005 and ^&^Significantly different from isotype CTRL.

GBex induced death in CD8^+^ Jurkat lymphocytes in a dose-dependent manner (IC_50_ = 4.2 µg) (Figure 3A) and induced apoptosis in primary activated human CD8^+^ T cells (Figure 3B). Blocking of FAS signaling with an anti-FasL Ab reversed this effect (Figure 3C). In addition, we observed a reduction in TNF-α and IFN-γ expression levels in CD8^+^ T lymphocytes (Figure 3D), while expression levels of IL-2 or CCL3 were not altered. Thus, GBex treatment inhibited activation of CD8^+^ T and NK cells, suppressed cytokine/chemokine production, and induced death of T lymphocytes.

### GBex Modulate Macrophage Polarization

**Figure 4.**
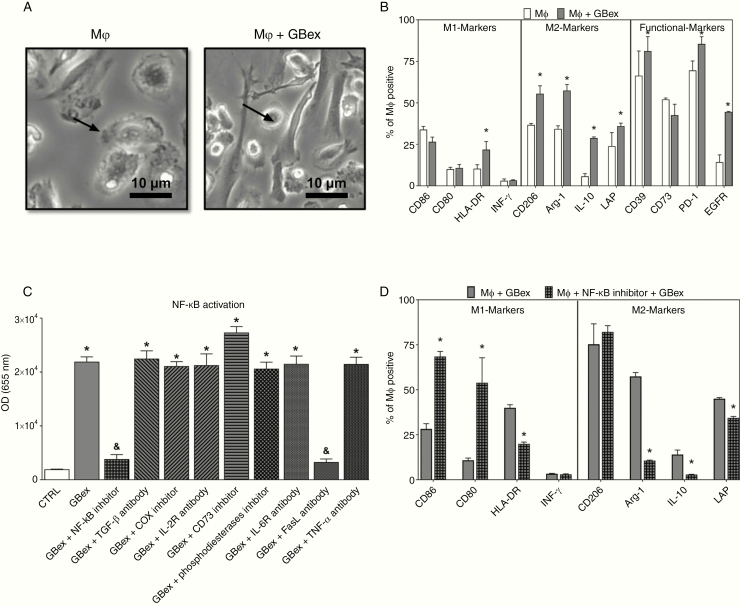
GBex induced M2-like polarization via NF-κB activation in macrophages. (A) Representative phase-contrast microphotographs of macrophages after treatment with GBex for 72 h. Note alterations of macrophage morphology in Mφ + GBex (bar equals 10 µm). (B) Macrophage polarization after treatment with GBex for 72 h. The panel shows M1 markers (CD86, CD80, HLA-DR, and INF-γ), M2 markers (CD206, Arginase-1, IL-10, and LAP), and functional markers (CD39, CD73, PD-1, and EGFR). (C) Biological activity of GBex (20 µg) on the activity of the NF-κB pathway macrophages. *Significantly different from CTRL and ^&^significantly different from GBex at P<0.05. (D) Macrophage polarization after treatment with the NF-κB inhibitor for 30 min followed by the addition of GBex. Values represent the mean ± SEM from 3 independent experiments. Data were analyzed by ANOVA followed by post hoc comparisons (Tukey–Kramer test). *Significantly different from macrophages+ GBex group at *P* < 0.05.

Human macrophages in primary cultures were exposed to GBex (25 ug/106 cells/mL) for 72 h. Macrophages cultivated alone in 10% RPMI served as controls. Macrophages were positive for CD68, CD71, and CD61 and were CD14^high^/CD16^low^ (96%) (Supplementary Figure 2). Following co-incubation with GBex, macrophages acquired the fibroblast shape (Figure 4A) and expressed M2 markers, including CD206, Arginase-1, IL-10, and LAP. In contrast, expression levels of M1 markers (CD86, CD80, and INF-γ) remained unchanged relative to no GBex control. In addition, macrophages co-incubated with GBex increased expression levels of CD39, PD-1, and EGFR (Figure 4B). Furthermore, GBex induced activation of the NF-κB pathway (11 folds) and blocking FasL signaling with anti-FAS mAbs (but not isotype control Ab) resulted in complete inhibition of NF-κB activation (Figure 4C). In macrophages, inhibition of the NF-κB pathway resulted in a reversal of the M2-like phenotype to the M1-like phenotype (Figure 4D). These data identify a new GBex-mediated suppression mechanism involving FasL-induced NF-κB activation and macrophages polarization.

To determine whether M2-like macrophages reprogramed by GBex enhance tumor proliferation, GB cell lines were cultured in macrophage CM, and the glioma cell viability was evaluated. CM from naïve macrophages did not promote GB cell growth (Supplementary Figure 3A). In contrast, CM from macrophages exposed to GBex increased GB cell viability. The data indicate that GBex-treated macrophages not only assume a more immunosuppressive phenotype but also acquire pro-tumor functions.

### GBex Activate Multiple Molecular Pathways in Macrophages and CD8^+^ T Cells

**Figure 5.**
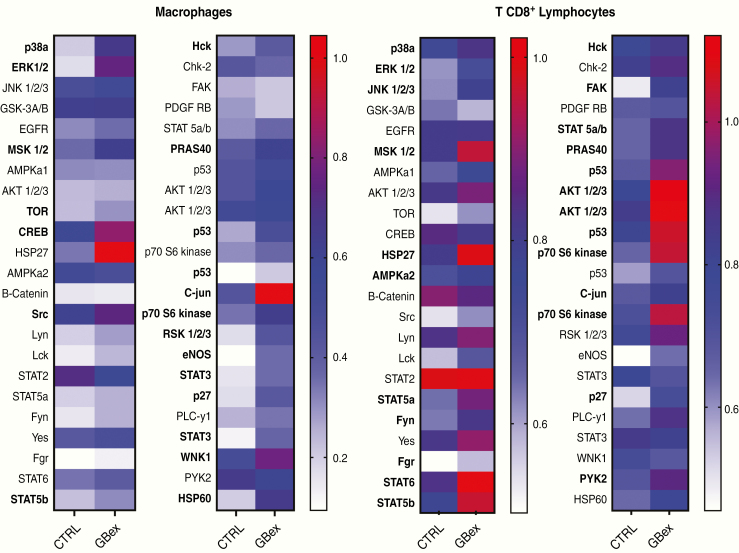
GBex-induced phosphorylation in multiple molecular pathways in macrophages and CD8^+^ T cells. Analysis of total proteins (300 μg) in lysates of macrophages or CD8^+^ T cells which were co-incubated with GBex for 15 or 12 min, respectively. Values are normalized to reference spots on the membranes. The arrays were quantified using ImageJ and the most important changes are highlighted in bold.

To further evaluate molecular mechanisms underlying the described GBex-mediated effects, changes in the cell signaling pathways in macrophages and CD8^+^ T cells co-incubated with GBex were evaluated. GBex (25 µg/2 × 10^6^ cells/1 mL) induced phosphorylation in the following macrophage pathways: p38a, ERK, MSK, TOR, CREB, Scr, STAT5b, Hck, PRAS40, p53, P70s6 kinase, RSK, eNOS, STAT3, p27, WNK1, HSP27, C-jun, and HSP60 (Figure 5 and Supplementary Figure 4). Co-incubation of CD8^+^ T cells with Gbex-induced phosphorylation of MSK, Scr, STAT5a, Fgr, STAT5b, STAT2, Hck, and PRAS40 (Figure 5 and Supplementary Figure 4). The data indicate that GBex simultaneously induced changes in phosphorylation of multiple molecular pathways in immune cells.

### Consequences of GBex Delivery to Normal Healthy Mice

**Figure 6.**
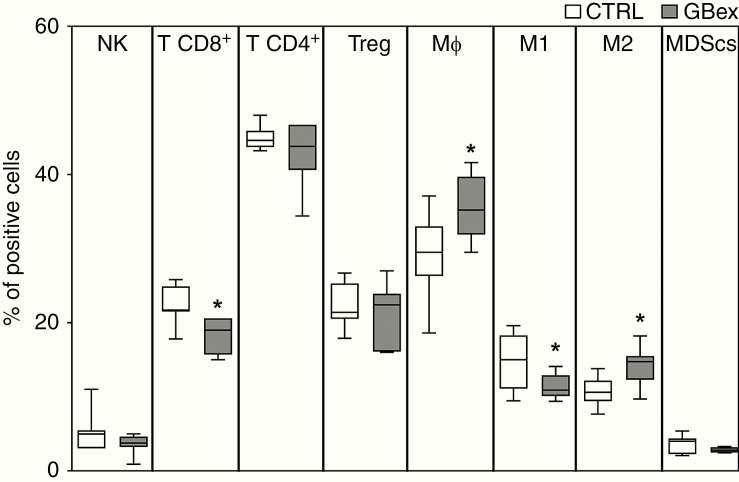
In vivo changes in immune cells in the spleen of normal mice injected intravenously with 1 mg/kg of GBex. Mice received intravenous injections of GBex in PBS (1 mg/kg or equivalent volume) every 3 days for 16 days (see the schedule in Supplementary Figure 1). Animals were sacrificed and spleens were harvested, dissociated to obtain a single-cell suspension. The frequency of immune cells was determined by immunostaining and flow cytometry. The percentages of NK (CD45^+^ NKp16^+^), CD8^+^ T cells (CD45^+^CD3^+^CD8^+^), CD4^+^ T cells (CD45^+^CD3^+^CD4^+^), T-regulatory cells (CD4^+^FOXP3^+^), macrophages (CD45^+^CD11b^+^F4/80^+^), M1-like macrophages (CD45^+^CD11b^+^CD80^+^CD86^+^), M2-like macrophages (CD45^+^CD11b^+^CD206^+^), and MDSCs (CD45^+^CD11b^+^Gr1^+^) were determined in the spleen. The values represent the mean values ± SEM from 7 animals. *Indicates a significant difference from the CTRL (*P* < .05) as determined by Student’s *t*-test.

Intravenous injections of GBex into normal mice reduced the frequency of T CD8^+^ and M1-like macrophages in the spleen, while increasing the number of total macrophages and M2-like macrophages (Figure 6). In addition, 2 weeks after GBex injections, reduced numbers of NK cells and M1-like macrophages, with increased total macrophage numbers, were observed in the blood of mice (Supplementary Figure 5). The in vivo data corroborate our in vitro results with human cells providing support for the observed GBex-mediated alterations of immune cells functions.

## Discussion

EVs, including exosomes, are used by tumor cells for transmission of messages to other cells not only in the local TME but also systemically.^15,29^ Upon release by tumor cells, exosomes are rapidly disseminated and can be recovered from all body fluids.^30^ EVs have been implicated in the reprogramming of the normal cells in TME to favor cancer progression.^15,31^ Among EVs, exosomes are considered to be ideal message carriers because of their small size, ubiquitous presence in all body fluids, and the ability to cross the BBB.^15,29,32^ The cargo of tumor-derived exosomes mimics that of the parent tumor cells^33^ and contains an excess of multiple immunoregulatory proteins which can modulate functions of adaptive and innate immune cells.^15,22^ As shown here, GBex contain an excess of immunosuppressive proteins that downregulate activation on CD4^+^ T cells and NK cells, reduce cytokine production and mediate apoptosis of CD8^+^ T cells, and promote the acquisition of the immunosuppressive phenotype in macrophages in vitro. Thus, GBex mediated varied and potent immunosuppressive in vitro activities.

Immunosuppressive activities of GBex were also evaluated in vivo in an immunocompetent mouse model. Naïve healthy mice were selected for these studies with the expectation of demonstrating in vivo depletion of normal immune cells in the periphery by GBex without apparent toxicity. As reported here, GBex *reduced* the number of effector immune cells and *increased* the number of M2 macrophages which support the tumor-permissive environment. This latter mechanism of polarization toward immunosuppressive macrophages may be especially relevant to brain tumors, which are enriched in macrophages/microglia (up to 30% of the tumor mass),^34^ and where GBex-mediated polarization might have a major impact on tumor progression. Although a physiological dose of exosomes is not known and, due to their abundant secretion and rapid clearance, is difficult to determine,^35^ we have previously shown that a single intravenous injection of tumor-derived exosomes (70–90 µg protein) enhanced carcinogenesis and suppressed immune cell functions in 4NQO mice.^36,37^ In our study, the optimal physiological dose of the injected GBex is likely to be governed by the rate of GBex production and release from the tumor and thus may be personalized. Nevertheless, similar growth-promoting and in vitro or in vivo immunosuppressive activities of tumor-derived exosomes were previously reported by others in several cancer types.^38–42^ Thus, immune suppression mediated by tumor-derived exosomes and extending beyond the local TME to the periphery emerges as a universal mechanism utilized by tumors to escape from the host immune system.

Several previous studies have examined GBex interactions with immune cells.^21–23^ Exosomes derived from GB stem-like cells were reported to enhance the expression of M2 markers in macrophages and to promote their phagocytic activity.^19^ In addition, GBex induced changes in the cytokine secretion profile of microglia, converting them to cells promoting GB growth and invasion, while suppressing functions of immune cells.^20^ Data in the literature also showed that exosomes derived from GB stem-like cells tend not to directly interact with T cells, but rather use myeloid cells to indirectly suppress lymphocyte proliferation and thus create the immunosuppressive TME.^23^ Despite the plethora of data, molecular mechanisms involved in interactions between GBex and different populations of human immune cells have not been fully elucidated. Examining molecular signaling involved in GBex interactions with immune cells, this study is the first to describe the coordinated *suppressive activity of many different signals simultaneously delivered by exosomes to target cells*. This type of molecular signaling by GBex leads to *simultaneous* activation of numerous molecular pathways in recipient cells, delivering a massive negative charge that induces a loss of functions in all subsets of immune cells.

Our data also identified a key role of NF-kB/FasL signaling in the GBex-mediated control of macrophage polarization and T-cell activation. FasL-mediated immunosuppression by tumor-derived exosomes was previously reported by our group.^43–45^ Here, we illustrate the FasL potential to activate the NF-kB pathway in macrophages. In addition, blocking IKKβ (a kinase necessary for activation of the NF-kB pathway) with a specific pharmacological inhibitor blocked the GBex-mediated conversion of M1 to M2 macrophages, which became more M1-like. In this context, NF-κB activation is essential for fostering the immunosuppressive phenotype of macrophages in the TME and thus may be a target for preventing M2-like macrophages from providing support for tumor growth.

In summary, our studies emphasize a major role of GBex in mediating reprogramming of immune cell numbers and functions in the TME and thus favoring the promotion of tumor progression. Our in vitro model was designed to mimic the glioblastoma TME. GBex were immunosuppressive in ex vivo functional assays and in vivo in mice, altering numbers/functions of all subsets of immune cells. The mechanism of GBex-mediated reprogramming involved the simultaneous delivery of numerous suppressive signals to immune cells which are known to reside in the TME. Specifically, Gbex-induced differentiation of macrophages into the M2-like cells creating a strongly immunosuppressive environment characteristic of TME and supportive of GB pathogenesis and progression.

## Funding

This work was supported by the National Institutes of Health (RO-I CA168628 and P306A047904 to T.L.W.); Programa de Doutorado Sanduíche no Exterior (PDSE) (88881.188926/2018-01 to J.H.A.) from Coordenação de Aperfeiçoamento de Pessoal de Nível Superior (CAPES); Leopoldina Fellowship (LPDS 2017-12 to N.L.) from the German National Academy of Sciences.

*Conflict of interest statement*. No potential conflict of interest was reported by the authors.

## Authorship Statement.

J.H.A.: Experimental design, GBex isolation and characterization, in vitro and in vivo functional assays, flow cytometry experiments, discussion of results, and manuscript preparation. N.L.: Western Blots, exosomes isolation, discussion of results, and figure preparation. S.Y.: Performed the reporter assay and obtained TEM images. A.R.: Staining and flow cytometry analysis with in vivo samples. E.B.: Discussion of results and manuscript preparation. T.L.W.: Financial support, supervision and interpretation of results, and manuscript preparation.

## Supplementary Material

vdaa056_suppl_Supplementary_Figure_1

vdaa056_suppl_Supplementary_Figure_2

vdaa056_suppl_Supplementary_Figure_3

vdaa056_suppl_Supplementary_Figure_4

vdaa056_suppl_Supplementary_Figure_5

vdaa056_suppl_Supplementary_Figure_6

vdaa056_suppl_Supplementary_Table_1

vdaa056_suppl_Supplementary_Figure_Legends
