# Supplementary figures and images for: Molecular profiles and immunomodulatory activities of glioblastoma-derived exosomes
Source: Neurooncol Adv. 2020 May 6;2(1):vdaa056. doi: 10.1093/noajnl/vdaa056 (PMC7262743; doi:10.1093/noajnl/vdaa056)

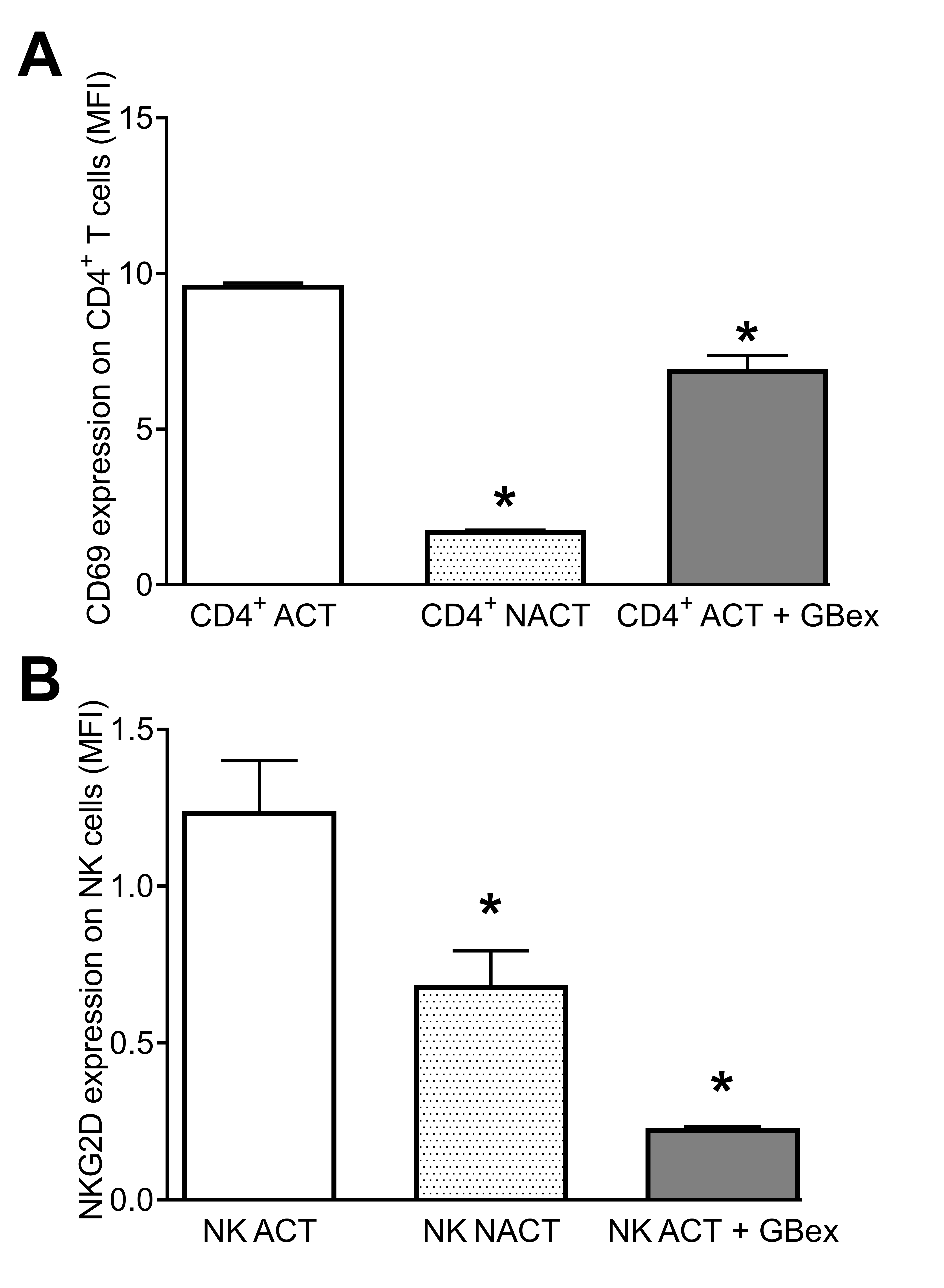

Supplement: vdaa056_suppl_Supplementary_Figure_1 [file vdaa056_suppl_supplementary_figure_1.png]

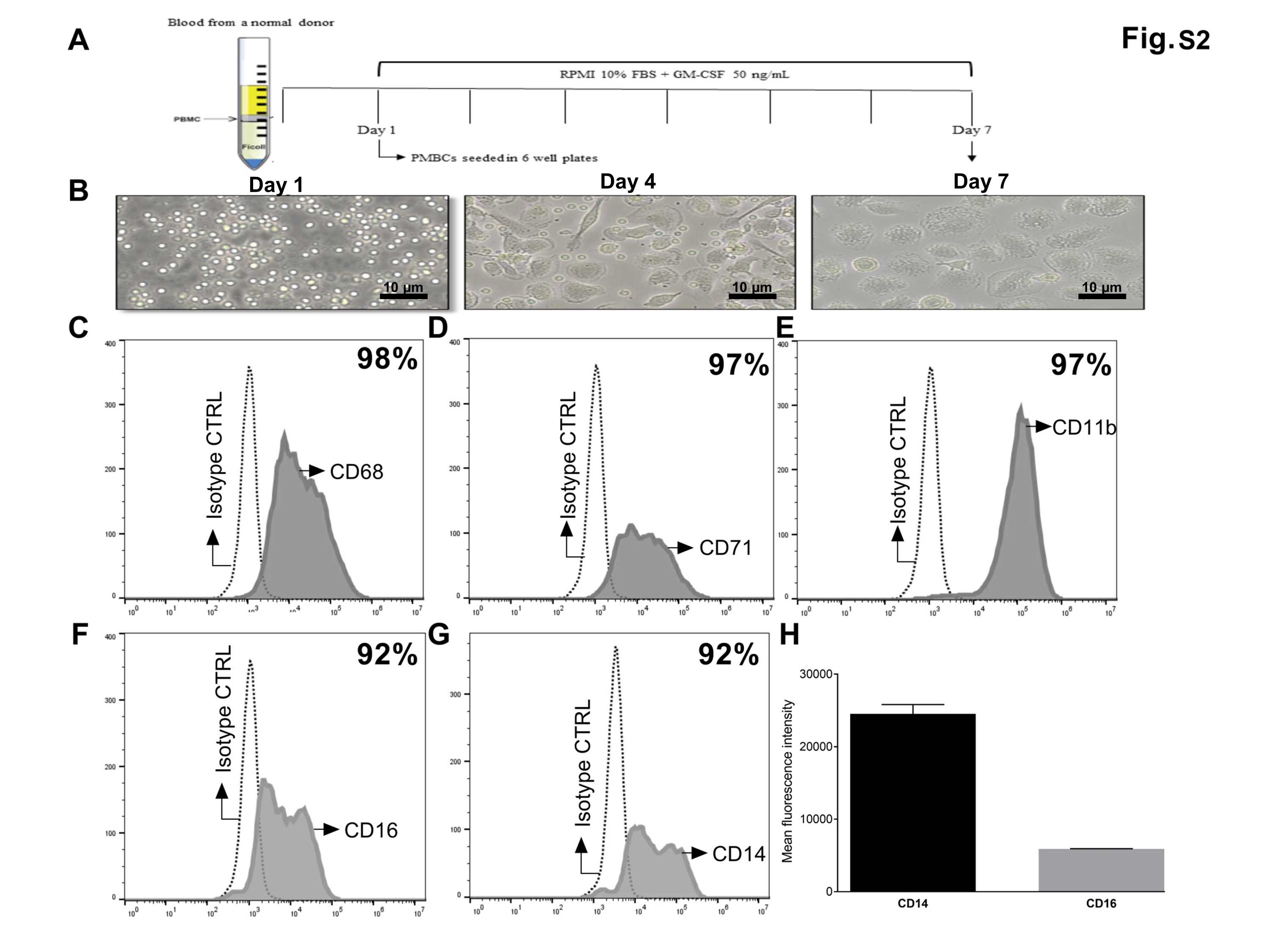

Supplement: vdaa056_suppl_Supplementary_Figure_2 [file vdaa056_suppl_supplementary_figure_2.png]

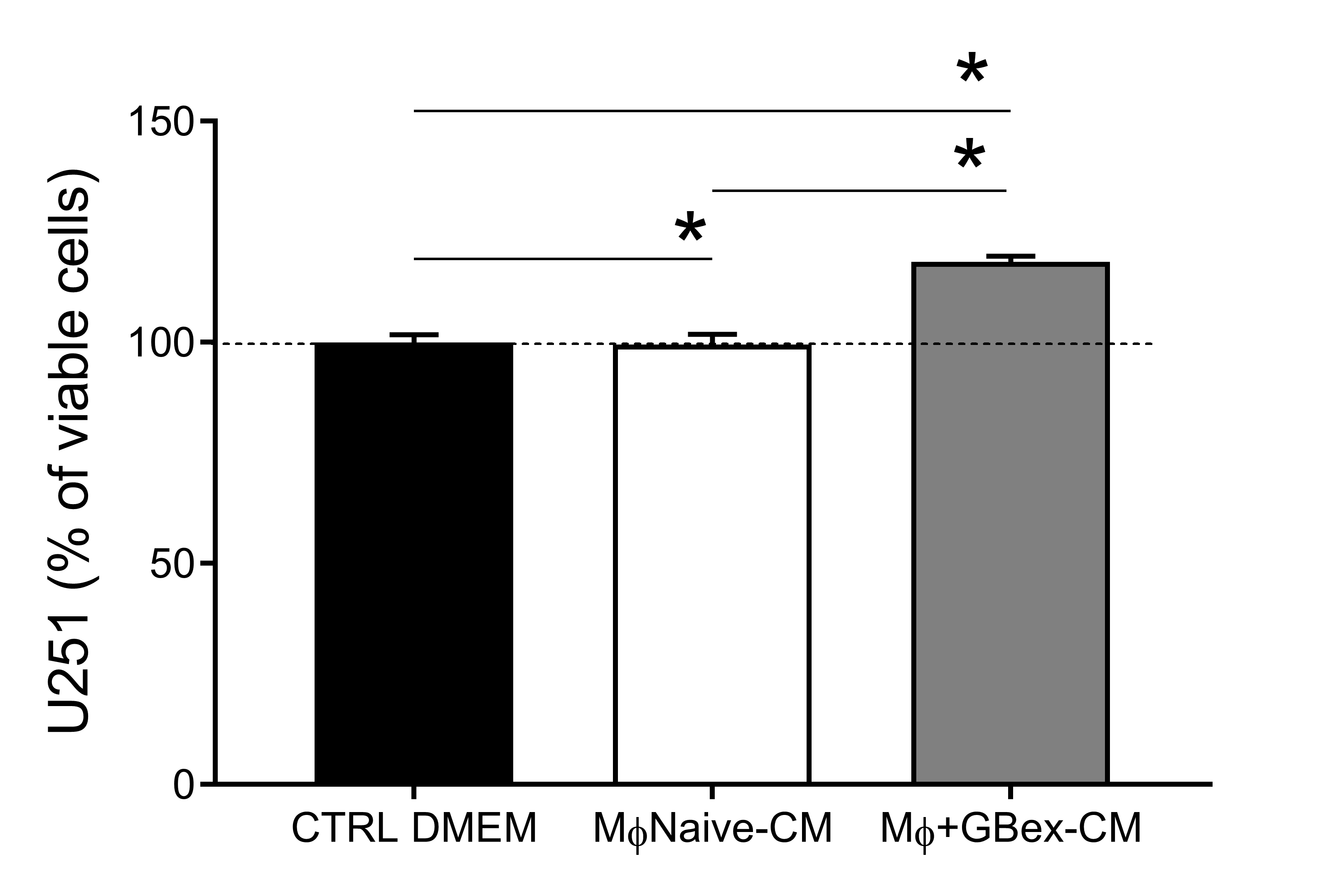

Supplement: vdaa056_suppl_Supplementary_Figure_3 [file vdaa056_suppl_supplementary_figure_3.png]

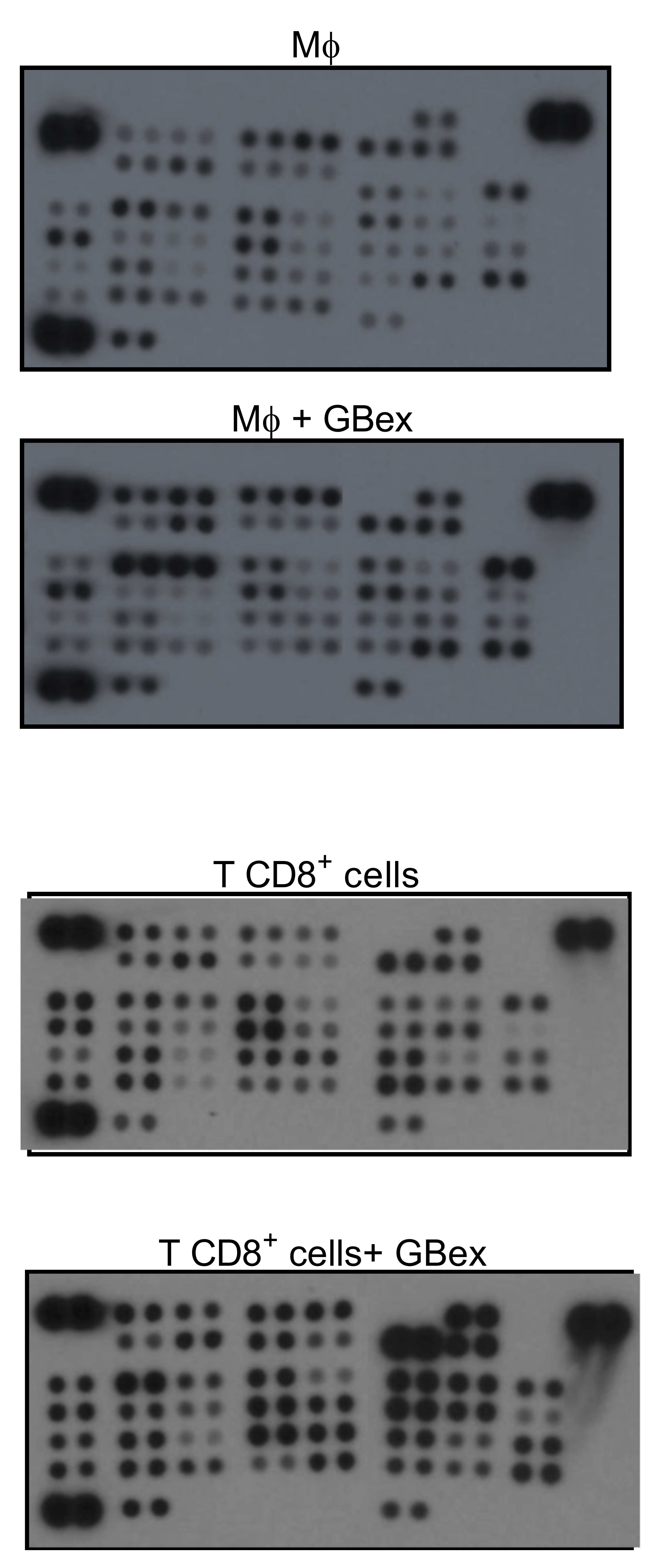

Supplement: vdaa056_suppl_Supplementary_Figure_4 [file vdaa056_suppl_supplementary_figure_4.png]

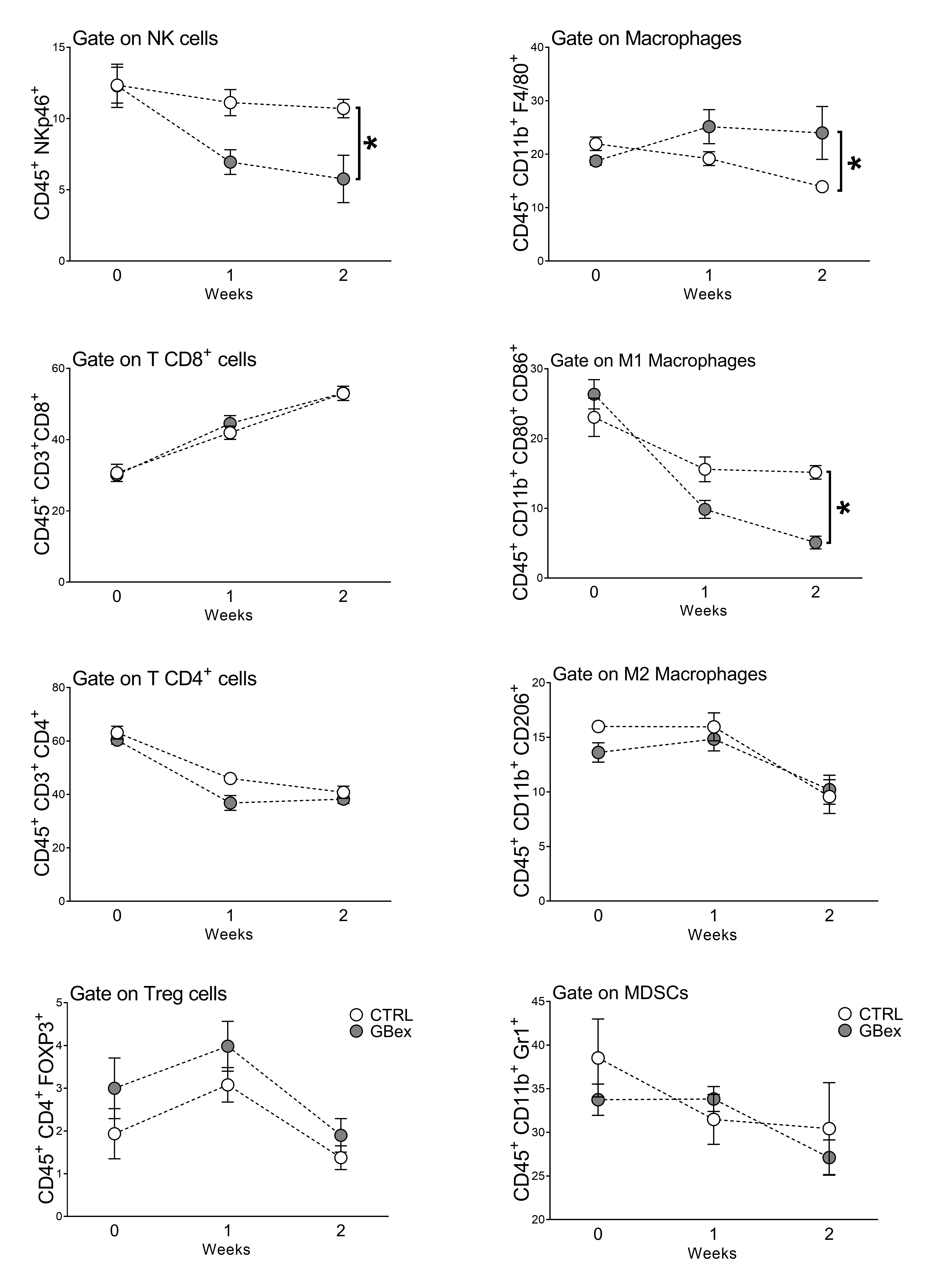

Supplement: vdaa056_suppl_Supplementary_Figure_5 [file vdaa056_suppl_supplementary_figure_5.png]

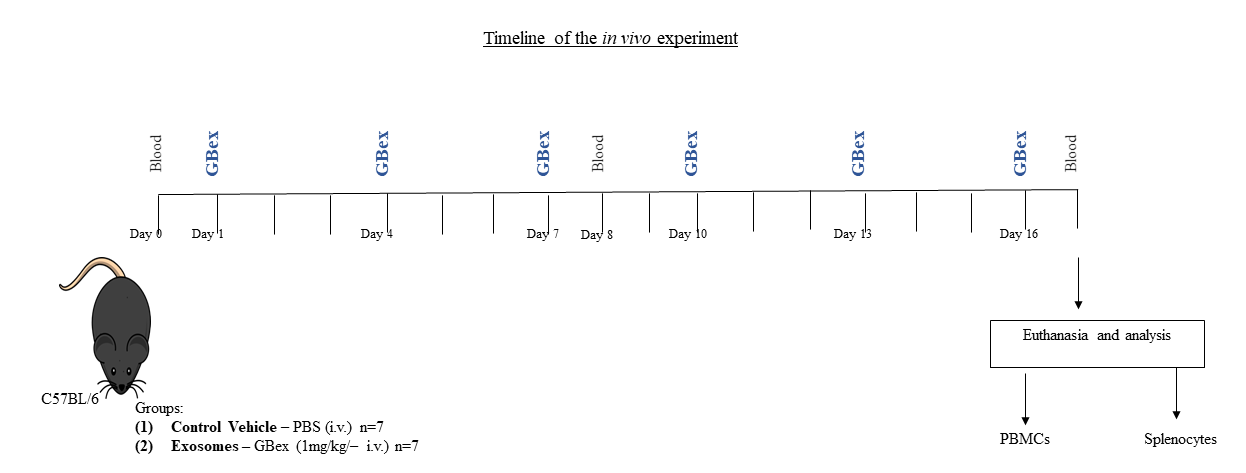

Supplement: vdaa056_suppl_Supplementary_Figure_6 [file vdaa056_suppl_supplementary_figure_6.png]
