# Supplementary material for: Molecular profiles and immunomodulatory activities of glioblastoma-derived exosomes
Source: Neurooncol Adv. 2020 May 6;2(1):vdaa056. doi: 10.1093/noajnl/vdaa056 (PMC7262743; doi:10.1093/noajnl/vdaa056)
Supplement: vdaa056_suppl_Supplementary_Table_1 [file vdaa056_suppl_supplementary_table_1.docx]

**Supplementary Table 1.**  List of antibodies and antibody dilutions used in the described experiments.

| **Antibody** | **Technique** | **Reference** | **Manufacturers** | **Dilution** |
| --- | --- | --- | --- | --- |
| ADA | WB | sc7450 | Santa Cruz | 1:1000 |
| Arginase-1 | WB | 98195 | Cell Signaling | 1:1000 |
| Arginase1-FITC | FC | IC5868F | R&D Systems | 1:5 |
| B7-1 | WB | sc376012 | Santa Cruz | 1:500 |
| CCL3-FITC | FC | IC2701F | R&D Systems | 1:50 |
| CD11b-Alexa fluor 647 | FC | 557686 | BD Bioscience | 1:50 |
| CD11b-PE | FC | PNIM2581U | Iotest | 1:3 |
| CD14-FITC | FC | 347493 | BD Bioscience | 1:50 |
| CD16-PE | FC | PNIM1238U | Iotest | 1:10 |
| CD206- PerCP/Cy5.5 | FC | 141716 | Biolegend | 1:50 |
| CD206-FITC | FC | 551135 | BD Bioscience | 1:3 |
| CD25-450 | FC | 48-0253-80 | eBioscience | 1:50 |
| CD25-PE | FC | 555434 | BD Bioscience | 1:3 |
| CD39 | WB | sc33558 | Santa Cruz | 1:400 |
| CD39‐APC | FC | 12-0399-42 | eBioscience | 1:10 |
| CD39-FITC | FC | 328206 | Biolegend | 1:25 |
| CD3-APC | FC | 47-0031-80 | Invitrogen | 1:50 |
| CD45- PE-Cy7 | FC | 25-0451-82 | Invitrogen | 1:50 |
| CD49b | FC | 17-5971-81 | Invitrogen | 1:50 |
| CD4‐FITC | FC | 11-0048-42 | Thermo Fisher | 1:10 |
| CD4-PE | FC | 555347 | BD Bioscience | 1:25 |
| CD4-PerCP-Cyanine5.5 | FC | 45-0042-80 | Invitrogen | 1:50 |
| CD68-PE | FC | 137013 | Biolegend | 1:50 |
| CD69-FITC | FC | 347823 | BD Bioscience | 1:25 |
| CD71-PE | FC | PNIM2001 | Iotest | 1:10 |
| CD73 | WB | sc25603 | Santa Cruz | 1:400 |
| CD73-FITC | FC | 344016 | Biolegend | 1:25 |
| CD80-BV510 | FC | 740130 | BD Bioscience | 1:50 |
| CD80-PE | FC | 557227 | BD Bioscience | 1:3 |
| CD8-645 | FC | 64-0081-80 | Invitrogen | 1:50 |
| CD86-BV786 | FC | 740877 | BD Bioscience | 1:50 |
| CD86-PE | FC | PNIM2729U | Iotest | 1:10 |
| CD9 | WB | ab65260 | Abcam | 1:500 |
| COX-2 | WB | ab52237 | Abcam | 1:500 |
| CTL-4 | WB | ab134090 | Abcam | 1:5000 |
| EGFR-APC | FC | 352906 | Biolegend | 1:10 |
| F4/80-eFluor450 | FC | 48-4801-80 | Invitrogen | 1:50 |
| Fas | WB | ab133619 | Abcam | 1:1000 |
| FasL | WB | #4273 | Cell Signaling | 1:1000 |
| Gr-1-Alexa Fluor 700 | FC | 56-5931-80 | Invitrogen | 1:50 |
| HLA-DR-FITC | FC | PNIMO436U | Iotest | 1:3 |
| IL-10-PE | FC | 559330 | BD Bioscience | 1:3 |
| IL-2-APC | FC | 341116 | BD Bioscience | 1:50 |
| INF-γ-APC | FC | 554702 | BD Bioscience | 1:10 |
| LAP-PE | FC | 349604 | Biolegend | 1:3 |
| Ly-6C- APC | FC | 47-5932-80 | Invitrogen | 1:50 |
| Ly-6G-PE/Dazzle 594 | FC | 127647 | Biolegend | 1:50 |
| MHCII-FITC | FC | 11-5321-81 | Invitrogen | 1:50 |
| NKG2D-PE | FC | PN A08934 | Beckman Coulter | 1:25 |
| NKp46-FITC | FC | 560756 | BD Bioscience | 1:50 |
| OX40 ligand | WB | ab108083 | Abcam | 1:500 |
| PD-1-PE | FC | 4345442 | Invitrogen | 1:10 |
| TNF-α | WB | 37075 | Cell Signaling | 1:1000 |
| TNF-α-PE | FC | 12-7321-82 | Thermo Fisher | 1:100 |
| TRAIL | WB | MA1-41027 | Invitrogen | 1:500 |
| TSG101 | WB | ab83 | Abcam | 1:500 |
| VISTA-PE | FC | 566269 | BD Bioscience | 1:50 |

Legend: WB: Western Blot; FC: Flow cytometry.
