# Supplementary material for: Molecular profiles and immunomodulatory activities of glioblastoma-derived exosomes
Source: Neurooncol Adv. 2020 May 6;2(1):vdaa056. doi: 10.1093/noajnl/vdaa056 (PMC7262743; doi:10.1093/noajnl/vdaa056)
Supplement: vdaa056_suppl_Supplementary_Figure_Legends [file vdaa056_suppl_supplementary_figure_legends.docx]

**Supplementary file**

**Supplementary Figure 1. GBex suppress activation of CD4^+^ T and NK cells.** In **(A),** flow cytometry data for GBex-mediated suppression of CD69 expression levels on the surface of human activated CD4^+^ T cells after co-incubation with GBex for 24h and in (**B)** GBex-mediated suppression of NKG2D-expression levels on the surface of human activated NK cells after co-incubation with GBex for 72h**.** Data are relative expression levels of CD69 on activated CD4^+^ T cells or activated NK cells (MFI relative to isotype controls). Data were analyzed by ANOVA followed by *post-hoc* comparisons (Tukey test). *significantly different from control cells at P<0.05. ACT: activated cells and NACT: not-activated cells.

**Supplementary Figure 2. Evaluation of macrophage differentiation in vitro**. In (**A**), the experimental protocol used for monocyte isolation. Blood obtained from a healthy donor was placed on a density gradient to separate PBMC. Monocytes were isolated by adherence to plastic and seeded into wells of a 6-well plates. Macrophage differentiation was stimulated using GM-CSF (50ng/mL). After 7 days, macrophages were analyzed by flow cytometry. (**B**) Representative photos of cells throughout the differentiation process (bar equals 10µm): (left) adhered monocytes immediately after the isolation; (middle) monocytes 96h after the beginning of the GM-CSF differentiation process; (right) monocytes already differentiated to macrophages after 7d of culture with GM-CSF. Isolated monocytes were analyzed by flow cytometry for the (**C**) CD68, (**D**) CD71, (**E**) CD11b, (**F**) CD14 and (**G**) CD16. (**H**) MFI for CD14 and CD16 markers.

**Supplementary Figure 3. Conditioned medium (CM) from macrophages after GBex treatment contain soluble factors which induced GB proliferation.** Macrophages were co-incubated with GBex for 72h. The conditioned medium was collected and added to the glioblastoma U251 cells. Cells cultured in DMEM medium for 72h (black bar), CM from naïve macrophages (white bar) and CM from macrophages after GBex treatment (grey bar). Cell viability was determined by MTS. Values represent the mean ± SEM from at least three independent experiments performed in triplicate. Similar results were obtained with GBex co-incubated with the two other glioblastoma cell lines. Data were analyzed by ANOVA followed by post hoc comparisons (Tukey-Kramer test). *significantly different at P<0.05.

**Supplementary Figure 4. GBex induced phosphorylation in several pathways in macrophages and T CD8^+^ cells.** The proteome profile arrays show the analysis of 300 μg total protein of macrophage cell lysate (upper array) or CD8^+^ T cell lysate (lower array) for levels of protein phosphorylation.

**Supplementary Figure 5.** Mice received IV injections of GBex in PBS (1 mg/Kg) every 3 days for 16 days (Fig. S1). Animals were sacrificed and blood was collected for PBMCs isolation.

The frequency of immune cells was determined by immunostaining and flow cytometry. The percentages of NK (CD45^+^NKp16^+^), T-CD8^+^ (CD45^+^CD3^+^CD8^+^), T-CD4^+^ (CD45^+^CD3^+^CD4^+^), T-regulatory (CD4^+^FOXP3^+^), macrophages (CD45^+^CD11b^+^ F4/80^+^), M1-like macrophages (CD45^+^CD11b^+^CD80^+^ CD86^+^), M2-like macrophages (CD45^+^CD11b^+^ CD206^+^) and MDSCs (CD45^+^CD11b^+^ Gr1^+^) were determined in PBMCs. The values represent the mean values± SEM from seven animals. * indicates a significant difference from the CTRL (p < 0.05) as determined by Student’s t test.

**Supplementary Figure 6. Timeline of the *in vivo* experiment.** **GBex *in vivo* delivery of normal mice changes the immune microenvironment.** Mice (C57BL/6) received IV injections of GBex in PBS (1mg/Kg or equivalent volume) every 3 days for 16 days. Animals were sacrificed and spleen were harvested. The blood was collected in the time points indicated in the figure and the PBMCs were isolated.
